# CSACI position statement: systemic effect of inhaled corticosteroids on adrenal suppression in the management of pediatric asthma

**DOI:** 10.1186/s13223-015-0075-z

**Published:** 2015-03-14

**Authors:** Karine Issa-El-Khoury, Harold Kim, Edmond S Chan, Tim Vander Leek, Francisco Noya

**Affiliations:** Division of Allergy and Immunology, McGill University, 2300 Tupper Street, C-510, Montreal, QC H3H 1P3 Canada; Department of Medicine, Division of Allergy and Immunology, Western University, London, ON Canada; Department of Medicine, Division of Allergy and Immunology, McMaster University, Hamilton, ON Canada; Department of Pediatrics, Division of Allergy and Immunology, University of British Columbia, Vancouver, BC Canada; Department of Pediatrics, Division of Clinical Immunology and Allergy, University of Alberta, Edmonton, AB Canada

**Keywords:** Asthma, Inhaled corticosteroids, Fluticasone, Adrenal suppression

## Abstract

Asthma is a chronic inflammatory disease of the airways that affects a growing number of children and adolescents. Inhaled corticosteroids (ICS) are the mainstay of treatment in persistent asthma, with a stepwise approach to increasing doses of ICS depending on asthma severity and control. ICS have known local and systemic side effects, of which adrenal suppression is still under-recognized. The latter is associated with chronic exposure and higher doses, although it has rarely been reported in children receiving low doses for a short period of time. The Canadian Society of Allergy and Clinical Immunology (CSACI) therefore recommends that physicians screen for adrenal suppression in children receiving high doses for more than 6 months and to consider screening those on medium dose if the risk is deemed higher by factors that increase an individual’s systemic corticosteroid exposure. Morning serum cortisol level can be used as a screening tool and abnormal results or normal results with a high index of suspicion should be confirmed with low-dose ACTH stimulation tests.

## Background

Asthma affects about 10% of the Canadian population, and 50-80% of children affected develop it before the age of 5 years [1]. It is a complex disorder with recurring but variable respiratory symptoms due to reversible airflow obstruction, bronchial hyperresponsiveness and underlying chronic inflammation; it causes significant morbidity and mortality, and is a major cause of health-related expenditures.

Topical anti-inflammatory therapy has become the main modality of asthma control therapy, in children, adolescent, and adults, with inhaled corticosteroids (ICS) being the preferred choice after four decades of clinical experience. As stated in the Canadian Thoracic Society Asthma Management Continuum 2010 Consensus, “there is robust evidence confirming that ICS therapy is effective at reducing asthma symptoms, improving health-related quality of life, improving lung function, decreasing airway hyper-responsiveness, controlling airway inflammation, reducing the frequency and severity of exacerbations, and reducing asthma mortality. Most patients can achieve asthma control using relatively low doses of ICS, which will produce maximum or near-maximum clinical benefit, with minimal risks of long-term adverse effects [2].”

A daily ICS regimen is recommended for the treatment of persistent asthma starting with low-dose monotherapy for most children and adolescents, with a step-up approach to medium and high-dose ICS (typically in conjunction with add-on therapy) depending on asthma severity and control. It is well established that the higher the daily dosage, the larger the probability of side effects will be [2,3]. In recent Canadian asthma guidelines [2,3] low, medium, and high ICS dose are defined, respectively, as ≤250, 251–500, and >500 μg /day for individuals >12 years of age and ≤200, 201–400, and >400 μg /day of fluticasone propionate (FP) or equivalent in those 6 to 11 years old. The dose equivalences of the different ICS products available in Canada are shown in Table 1.

**Table 1:** Dose Equivalences of inhaled corticosteroids available in Canada*

**Product**	**Low Dose**	**Medium Dose**	**High Dose**
Beclomethasone (Qvar™)	≤250 μg	>250-500 μg	>500 μg
	*(*≤*200* μg)**		*(*≥*400* μg)**
Budesonide (Pulmicort™)	≤400 μg	>400-800 μg	>800 μg
(Via inhaler)			
Fluticasone (Flovent™)	≤250 μg	>250-500 μg	>500 μg
	*(*≤*200* μg)**		*(*≥*400* μg)**
Ciclesonide (Alvesco™)	≤200 μg	>200-400 μg	>400 μg
Mometasone (Asmanex™)	≤200 μg	>200-400 μg	>400 μg

*Adapted from reference 3.

**Age < 12 y.

Bioavailability, determined by dosage, device used, particle size, lung versus upper airway deposition, proportions of inactive pro-drug versus active drug at mucosal surfaces, as well as serum protein binding, all influence the probability of experiencing side effects from the use of ICS [4].

Local ICS side effects include oral candidiasis and dysphonia, usually prevented by rinsing after inhalation and use of a spacer in children on metered dose inhalers. Systemic side effects include adrenal suppression, decreased bone mineralization, and growth suppression [5]. Of these, a very important yet under-recognized potential side effect is adrenal suppression, also referred to as hypothalamic-pituitary-adrenal (HPA) axis suppression. This suppression occurs secondary to the negative feedback exerted by exogenous corticosteroids on the HPA axis. Chronic exposure and higher doses can lead to HPA axis deficiency, atrophy of the adrenal glands and adrenal suppression with subsequent inability to produce sufficient amount of cortisol. This can persist for up to one year after discontinuation of treatment The clinical presentation of adrenal suppression is referred to as adrenal insufficiency, and ranges from non-specific and unrecognized symptoms like fatigue, headache, abdominal pain, to more concerning manifestations as generalized weakness, poor growth, syncope, hypotension, hyponatremia, and hypoglycemia [6,7].

Lipworth et al [8] reviewed the literature on systemic adverse effects of ICS and found evidence of HPA suppression in children and adults on doses >750 μg/day of FP (i.e. high dose ICS as defined above). Smith et al [9] reported HPA axis suppression in 9.3% of children who received medium or lower dose ICS for their age (200 to 500 μg/day). In this latter study, 20% of the children had received oral prednisone within the preceding year and less than 10% were receiving intranasal steroids. Among those receiving low dose ICS, the duration of treatment ranged from 3 to 24 months. They also correlated a 250 μg/day increase in dose to a 50% increase in the odds of HPA axis suppression. Others have reported adrenal suppression in 35% to 65% of children on ICS ≥500 μg/day for up to 16 weeks after starting therapy [10-14]. Furthermore, if an individual develops adrenal suppression, then a subsequent sudden discontinuation of steroid treatment can lead to adrenal crisis. This can also occur in circumstances of significant stress such as surgery or illness. In a British survey published in 2002 [7], 28 children were identified who experienced adrenal crisis. The children described in this paper were between 3.3 and 10 years of age (mean 6.4 years old) and 27 of these children were taking a high dose of FP (mean 980 μg/day, range 500–2000 μg/day).

### Screening for adrenal suppression

Based on these findings, the Canadian Society of Allergy and Clinical Immunology (CSACI) recommends that all children and adolescents who are receiving a high dose of ICS as defined by the Canadian asthma guidelines [2,3] for 6 months or more be screened for adrenal suppression. Currently, there is not enough evidence to support the systematic screening of all children and adolescents on medium doses, but screening should be a consideration if the risk is deemed higher by factors that increase an individual’s systemic corticosteroid exposure. These factors include ICS dose in the higher end of the range, prolonged duration of treatment at such dose, concomitant use of nasal and topical corticosteroids, recent or frequent short courses oral steroids, high level of adherence to therapy, and smaller body mass for age [14]. Additionally, all children and adolescents who exhibit signs and symptoms that are suggestive for adrenal insufficiency (including poor growth), and regardless of ICS dose, should be screened.

The preferred initial method of screening for adrenal suppression is to measure morning serum cortisol level in children over 2 years of age who have established a circadian rhythm of cortisol secretion, and this test is readily available to physicians in Canada. Normal values vary per laboratory. For an accurate measurement, the morning serum cortisol level should be obtained following the specifications of your local laboratory.

Of note, a potential pitfall of this screening tool is that its sensitivity is about 60%, so normal values do not rule out HPA axis suppression [15]. Therefore, all those with abnormal results, as well as those with normal results and a high index of suspicion will require further assessment. It is advised that these children be referred to a paediatric endocrinologist for further assessment and co-management. The gold standard to measure HPA axis suppression is metyrapone testing, but this test is neither practical nor available in most centers. A more commonly used test is the ACTH stimulation testing. The latter previously used a conventional dose of 250 μg cosyntropin (a synthetic ACTH derivative), which was considered supraphysiological [7] and led to false-negative results. A meta-analysis [16] found that ACTH stimulation using a low-dose 1μg cosyntropin followed by serum cortisol measurement at 15 min, 30 min and 60 min was better for diagnosis. A peak cortisol level >500 nmol/L is normal, whereas a level <500 nmol/L is diagnostic of adrenal suppression. Prior to the test, patients need to withhold their exogenous ICS for 24 hours.

### Management of adrenal suppression

Patients with adrenal suppression should be managed in conjunction with an endocrinologist. Hydrocortisone at a physiologic dose (8–10 mg/m^2^/day) might be necessary until the morning serum cortisol returns to normal, usually after 6 to 12 months. In those in which the diagnosis was made by stimulation testing because the morning serum cortisol was normal, the endocrinologist should assess when the child is no longer considered to have adrenal suppression and no longer needs physiologic replacement therapy or stress precautions [6]. Patients and their families should be given written instructions on the need for systemic steroids at time of stress. The use of a medical identification tag is advised until the adrenal suppression resolves.

### Prevention of adrenal suppression

A majority of children and adolescents with asthma respond to low-dose ICS. Current Canadian asthma guidelines [2,3] recommend using the lowest effective ICS dose to control asthma symptoms, with subsequent use of add-on therapy with long-acting beta-2 agonists (LABA) in children >12 years old or increasing to medium ICS dose in those 6–11, when control is sub-optimal. Other factors for failure to achieve control, such as poor adherence, poor inhaler technique, untreated concomitant rhinitis/rhinosinusitis, and continued exposure to relevant allergic and nonallergic triggers, each need to be assessed and addressed before considering escalating asthma therapy. The most common reason for not achieving control is inadequate adherence to the prescribed regimen. Persistent rhinitis/rhinosinusitis are frequently present in children or adolescents with asthma and may cause a bothersome cough, including nocturnal cough, which may lead the practitioner to increase the dose of ICS. Adequate treatment of the upper airway not only improves upper airway symptoms but also decreases bronchial hyperreactivity [17]. If asthma treatment escalation is necessary, consultation with an asthma specialist is strongly recommended. It is important to inform patients and their families about the potential development of adrenal suppression when the patient is treated with high dose ICS; or with medium dose in those patients the clinician deems to have a higher risk because of the factors mentioned above. Regular follow-up is crucial to ensure appropriate screening for adrenal suppression and treatment when necessary.

### Key points

1. Daily inhaled corticosteroids are indicated for the treatment of persistent asthma in children; most patients respond to low-dose ICS (≤200 μg/day of FP or equivalent). ICS may be increased to medium dose in children 6–11 years old or LABA added in those >12 years of age if asthma is severe or poorly controlled. This step up should be done only after assessing that lack of control is not due to inadequate treatment adherence, poor inhaler technique, or co-morbidities such as uncontrolled upper airway disease, or that the diagnosis is other than asthma. In patients failing to respond to escalation, consultation with an asthma specialist is strongly recommended.
2. Systemic side effects of inhaled corticosteroids include HPA deficiency leading to adrenal suppression, usually associated with high doses and long treatment duration. It has been rarely reported in children receiving low or medium dose ICS for a short period of time.
3. Screening for adrenal suppression is recommended in children taking high dose ICS (≥500 μg/day of FP or equivalent; ≥400 μg/day under age 12) for more than 6 months. Consideration should be made to screen those on medium dose (251–500 μg/day of FP or equivalent; 201–400 μg/day under age 12) if the risk is deemed higher by factors that increase an individual’s systemic corticosteroid exposure. High-risk factors include ICS dose in the higher end of the range, prolonged duration of treatment at such dose, concomitant use of nasal and topical corticosteroids, recent or frequent short courses oral steroids, high level of adherence to therapy, and smaller body mass for age*.*
4. Assessment of morning serum cortisol is readily available but has poor sensitivity. Abnormal results or normal results with high index of suspicion should be referred to endocrinologist and adrenal suppression confirmed with a low-dose ACTH stimulation test.
5. When adrenal suppression is confirmed, children should be managed in conjunction with a pediatric endocrinologist. Oral physiologic corticosteroid replacement therapy should be prescribed and written instructions for stress corticosteroid dosing should be provided until the adrenal suppression resolves. The use of a medical identification tag is advised until the adrenal suppression resolves.

## Abbreviations

ICS: Inhaled corticosteroids
CSACI: Canadian Society of Allergy and Clinical Immunology
FP: Fluticasone propionate
HPA: Hypothalamic-pituitary-adrenal
LABA: long-acting beta-2 agonists
